# Comparison of freshly cultured versus freshly thawed (cryopreserved) mesenchymal stem cells in preclinical in vivo models of inflammation: a protocol for a preclinical systematic review and meta-analysis

**DOI:** 10.1186/s13643-020-01437-z

**Published:** 2020-08-19

**Authors:** Chintan Dave, Andrea McRae, Emily Doxtator, Shirley H. J. Mei, Katrina Sullivan, Dianna Wolfe, Josee Champagne, Lauralyn McIntyre

**Affiliations:** 1 Department of Medicine, The Ottawa Hospital, Ottawa, ON Canada; 2 Sinclair Centre for Regenerative Medicine, Ottawa Hospital Research Institute, Ottawa, ON Canada; 3 Knowledge Synthesis Group, Ottawa Hospital Research Institute, Ottawa, ON Canada; 4 Division of Critical Care, Department of Medicine, The Ottawa Hospital, Ottawa, ON Canada

**Keywords:** Preclinical systematic review, Cryopreserved, Thawed, Fresh, Cultured, Mesenchymal stromal cells (MSCs), Animal model

## Abstract

**Background:**

Mesenchymal stem cells (MSCs) are multipotent cells that demonstrate therapeutic potential for the treatment of acute and chronic inflammatory-mediated conditions. Especially for acute conditions, it is critical to have a readily available freshly thawed (cryopreserved) MSC product for rapid administration. Although controversial, some studies suggest that MSCs may lose their functionality with cryopreservation which in turn could render them non-efficacious.

**Objective:**

In controlled preclinical in vivo models of inflammation, to determine if there are differences in surrogate measures of preclinical efficacy between freshly cultured and freshly thawed MSCs

**Methods/design:**

A systematic search for pre-clinical *in vivo inflammatory model* studies will compare freshly cultured to freshly thawed MSCs from any source. The primary outcomes will include measures of in vivo preclinical efficacy; secondary outcomes will include measures of in vitro MSC potency. Electronic searches for MEDLINE and EMBASE will be constructed and reviewed by the Peer Review of Electronic Search Strategies (PRESS) process. If applicable, study outcomes will be meta-analyzed using a random effects model. Risk of bias will be assessed by the SYRCLE “Risk of Bias” assessment tool for preclinical in vivo studies.

**Discussion:**

The results of this systematic review will provide translational scientists, clinical trialists, health regulators, and the clinical and public community with the current pre-clinical evidence base related to the efficacy and potency of freshly cultured versus freshly thawed MSCs, help identify evidence gaps, and guide future related research.

**Systematic review registration:**

Protocol is submitted to PROSPERO for registration (pending confirmation) and will be submitted to Collaborative Approach to Meta-Analysis and Review of Animal Data from Experimental Studies (CAMARADES) for public posting.

## Background

Since the first transplantation of stem cells in 1957, research has heavily focused on hematopoietic stem cells (HSCs), which have now become standard-of-care for many indications [1]. The success of these transplantations established a new paradigm for stem cell therapy in the past 30 years. In recent years, studies have found that mesenchymal stem cells (MSCs) have functions that are unique to traditional stem cell therapies related to immunomodulation, and without the need for engraftment into tissues [2]. Since the early 2000s, reports of immunomodulatory properties of MSCs from the bone marrow were emerging [3–5], and these were reproducible in various species and models, including human case reports, that found in vitro-cultured MSCs had immunosuppressive function [6–8]. These results were also reproduced in MSCs from sources other than the bone marrow [9, 10]. Furthermore, numerous animal model and translational studies have reported capacity of MSCs to home to sites of injury/inflammation and carry out their immunomodulatory function, which adds to their attractiveness for use in the clinical setting [11]. As of June 2020, there were 885 MSC-related clinical trials registered on the NIH Clinical Trial Database. An accumulation of small clinical trials are now published that have tested MSCs in a variety of clinical trials for heterogeneous inflammatory-mediated conditions, examples include multiple sclerosis [12], graft-versus-host disease [13–15], osteoarthritis [16–20], inflammatory bowel disease (IBD) [21, 22], various pulmonary inflammatory diseases [23–25], and septic shock [26]. The limitation with many of these studies and in future real-world therapeutic applications of MSCs is the logistical and financial challenges that come from isolating, culturing, and cryopreserving MSCs. The majority of preclinical MSC research have examined the use of freshly cultured MSCs. However, this approach may not be feasible for acute indications, such as septic shock, where an off-the-shelf MSC treatment is desired. There have been conflicting studies regarding the effect of cryopreservation on MSCs and their functionality. Some studies show that freshly cultured MSCs are more potent in an in vitro setting when compared to their cryopreserved counterpart [27, 28], whereas other studies demonstrate that cryopreservation does not negatively impact the potency or efficacy of MSCs [29–32]. Given this uncertainty, a review of the preclinical literature is necessary.

To begin to address this evidence gap, our team will conduct a systematic synthesis of all preclinical in vivo studies that examine surrogate measures of in vivo efficacy of freshly cultured and freshly thawed MSCs in animal models of inflammation. The results of this systematic review will provide translational scientists, clinical trialists, health regulators, and the clinical and public community with the current pre-clinical evidence base related to the efficacy and potency of freshly cultured versus freshly thawed MSCs, and help identify evidence gaps to guide future related research.

### Study question

In controlled preclinical in vivo models of inflammation, are there differences in surrogate measures of preclinical efficacy between freshly cultured and freshly thawed MSCs?

## Methods/design

### Protocol and registration

The systematic review protocol was developed through discussions with our team of clinical (LM, CD) and preclinical research scientists (LM, SM, AM), an information specialist (AM), and experts in knowledge synthesis and translation (LM, AM, JC, DW). This systematic review will be conducted in consultation with the Cochrane Handbook and reported in accordance with the Preferred Reporting Items for Systematic Review and Meta-Analysis (PRISMA) guidelines [33, 34]. A copy of the PRISMA checklist will be provided as part of the supplementary documentation with the final report. The protocol will be registered within the International Prospective Register of Systematic Reviews (PROSPERO) and with the Collaborative Approach to Meta-Analysis and Review of Animal Data from Experimental Studies (CAMARADES) group.

### Study eligibility

#### Study design

Preclinical studies of in vivo models of inflammation that compare freshly cultured to freshly thawed MSC products (randomized, quasi-randomized, and non-randomized) will be included. Studies that only describe in vitro experiments comparing freshly cultured to freshly thawed MSC products will be excluded.

#### Population (preclinical model)

The population will include animal model of acute or chronic inflammation. These animal models (Table 1) may be diverse given that there are many human clinical diseases or conditions that are associated with inflammation [35–37]*.* Inflammation is the response, through the innate and adaptive immune system and inflammatory mediators, of the host to harmful stimuli, such as pathogens, cell damage, or irradiation. A series of coordinated and shared mechanisms and pathways of inflammation contribute to diverse clinical presentations [38]. Consequently, studies will be considered as models of inflammation if they are designed to mimic a clinical condition or syndrome that is associated with an inflammatory process. Studies that include immunocompromised animals (SCID) or treatments to immunosuppress the animals or that focus on the effects of MSCs on implantation and tissue regeneration (e.g., bone regeneration) will be excluded.

**Table 1:** Common examples of preclinical in vivo models of inflammation that have been used to assess in vivo markers of pre-clinical efficacy and/or in vitro MSC potency

Models of inflammation	
Sepsis	
Acute lung injury	
Inflammatory airway disease	
Ischemia-reperfusion injury	
Acute and chronic arthritis	
Chronic kidney disease	
Hindlimb ischemia	
Closure of acute and chronic skin wounds	

#### Intervention and comparison

Both the intervention and comparison groups will include the administration of MSCs that are derived from any MSC origin (bone marrow, adipose tissue, umbilical cord, or other) and source (xenogeneic, syngeneic, autologous, or allogeneic). MSCs must be administered during or after the induction of the experimental pre-clinical model (i.e., studies evaluating the preventive effects of MSCs will be excluded). All MSC delivery routes will be included. All differentiated MSCs (e.g., differentiated into a myocyte), mesenchymal progenitor cells (MPCs), mononuclear cell (MNC) fraction, and any stem cells that are not described as MSCs will be excluded. Non-MSC comparison groups (e.g., phosphate-buffered saline, fibroblasts) will also be described to examine the magnitude of the effect for the proposed outcomes (surrogate markers of preclinical in vivo efficacy and in vitro potency).

The intervention group will include freshly thawed MSCs. To be considered freshly thawed, MSCs need to be cryopreserved for any duration of time and be placed in culture for less than 24 h post-thaw prior to use in the given experiment. The comparison group will be freshly cultured MSCs. MSCs will be considered freshly cultured when the cells are either in continuous culture or cryopreserved but then thawed and placed in culture for at least 24 h prior to use in experiments. We used this 24-h culture time as a cutoff as previous experiments have demonstrated that cryopreserved MSCs may require 24 h of culture to recover their functionality [39].

MSCs that are pre-treated, pre-conditioned, genetically altered, or co-administered with other experimental interventions will be included if the same alteration is applied to both the freshly cultured and freshly thawed MSCs.

#### Outcomes

The primary outcome measures are surrogate measures of in vivo preclinical efficacy that are relevant to specific acute and chronic inflammatory animal models and are defined by two outcome domains: (1) organ dysfunction and composition of tissues (e.g., histopathological damage) and (2) protein expression and secretion (e.g., cytokine levels, immunohistochemistry analysis).

Our secondary outcomes include the measures of in vitro MSC potency (that may be described as additional experiments in the included in vivo studies). Ideally, potency should represent the MSCs’ mechanism of action; however, MSCs have complex and multiple mechanisms of action, all of which are not yet fully characterized or reported [40]. The International Society for Cellular Therapy has recently published a perspective paper on this topic [40] and suggest assessing MSC potency based on an assay matrix, namely, a collection of assays. These include a combination of in vitro analytical and/or biological assays. Examples of analytical in vitro assays include an assessment of the cellular secretome by ELISA (enzyme-linked immunosorbent assay) or of functional cell-based assays (in vitro assay culturing MSCs with responder immune cells). Hence, the two main secondary in vitro potency outcome domains include (1) co-culture assays and (2) protein expression and secretion.

#### Information sources

Search strategies will be developed and tested through an iterative process by an experienced medical information specialist in consultation with the research team. Six target articles provided by an expert in the field of preclinical research (SM) that were known prior to the development of the search strategy have been included in the search criteria to help capture all potential studies. Using the Ovid platform, Ovid MEDLINE®, OvidMEDLINE® In-Process & Other Non-Indexed Citations, and Embase Classic plus Embase will be searched. Additional searches will be performed on BIOSIS and Web of Science using Web of Knowledge. The strategy will be reviewed by another senior information specialist using the Peer Review of Electronic Search Strategies (PRESS) template. Search strategies will use a combination of controlled vocabulary (for example, mesenchymal stem cells, cryopreserved, acute or chronic inflammation) and keywords (for example, MSCs, cryopreservation, freshly-thawed, fresh). Vocabulary and syntax will be adjusted across the databases. No additional filters will be employed to ensure the largest number of relevant studies are captured. There will be no language or date restrictions on any of the searches. The proposed preliminary search strategy with all studies included until June 20, 2019, appears in Supplemental Table 1. The bibliographies of included studies and pertinent reviews will also be hand searched for further relevant studies.

### Study selection

The titles and abstracts of the search results will be screened independently by two investigators (CD, ED). The full-text of all potentially eligible studies will be retrieved and reviewed for eligibility, independently, by two members of the team using the a priori inclusion criteria described above. Disagreements between reviewers will be resolved by consensus or by a third member of the systematic review team (LM, SM).

### Data collection and process and data items

Data will be extracted independently by two members of the research team into standardized, pilot-tested Excel sheet forms. For all relevant outcomes, all variables of the in vivo and in vitro experiments will be collected, including control groups employed for each experiment/assay. Moreover, all details surrounding the cryopreservation process and the setup of the above experiments will be collected to understand any heterogeneity in cryopreservation and culture processes. Mean and standard deviation will be collected from the available data; if these are not available, the data provided from the study will be collected. MSCs will be characterized using the International Society of Cellular Therapy (ISCT) consensus statement (e.g., adherence to plastic, tri-lineage differentiation), but this will not be used as strict inclusion criteria. Specific data elements are listed in Table 2. The research team will contact authors of included studies for clarifications and to obtain additional information relevant to this review as appropriate.

**Table 2:** Data collection elements

Category	Specific items
Study characteristics	Study title, author, date of publication, journal published, country of publication, randomization process
Study population (animal model)	Animal type, age, gender, strain, and weight, presence of co-morbid illnesses
Model of inflammation and method of induction	Acute or chronic, direct or indirect infection, chemical-induced injury, ischemia-reperfusion, surgically induced model, details of model induction process
Intervention and comparison	International Society for Cellular Therapy criteria, MSC tissue source, MSC characteristics (including method of sorting), time and route of MSC administration, description of preparation and suspension of MSCs and controls (including medium used for cells, duration of culture, passage number, concentration/amount of MSCs used), vehicle used for delivery, storage conditions, cryopreservation methods (duration of cryopreservation, method used to cryopreserved MSCs, and concentration of MSCs during cryopreservation, and medium used to cryopreserve and whether any additives were used), post-thaw washing procedure and time to use in experiment, time from disease induction to MSC administration, MSC viability, surface marker expression
Co-interventions	Antibiotics, cytokines, cultured medium, membrane/scaffold system employed
Potency	MSC secretome and release assay, MSC effect on various cells, and PBMC proliferation co-culture assay
Surrogate markers of efficacy	Preclinical outcomes that are relevant as per the model of inflammation employed and fall within the two domains of organ dysfunction and protein expression and secretion: examples include arterial oxygenation, lung compliance, neovascularization, epithelialization, wound contraction rate, arthritic index, histological lung injury, etc.
Other	Presence of a priori sample size calculation. Industry sponsorship

*MSC* mesenchymal stromal cell

### Assessment of risk of bias

Risk of bias will be assessed independently by two reviewers (CD and AM), and disagreements will be resolved via consensus or by a third reviewer when necessary. All studies will be assessed as having high, low, or unclear risk of bias for the 10 domains of bias adapted from the SYRCLE “Risk of Bias” assessment tool for preclinical in vivo studies [41]. This tool has been adapted from the Cochrane Collaboration Risk of Bias tool employed in clinical studies, with the aim to incorporate key elements that are relevant for in vivo animal studies. A table of the 10 risk of bias domains is provided in the Appendix (Table 3). To date, no validated tool is available for the evaluation of risk of bias for in vitro studies, which is mainly driven by poor reporting of in vitro studies, limiting the assessment of internal validity, and the complexity/non-reproducible nature of many in-vitro experiments [42]. However, an adaptation based on the Cochrane Risk of Bias Tool and previous risk of bias assessments for in vitro systematic reviews to describe risk of bias elements that are relevant to in vitro studies is under development by our group [43–45].

### Data analysis

Given the broad range of models of inflammation that are likely to be included, the ability to conduct quantitative analyses may be limited. Thus, much of the synthesis may be qualitative but supported by quantitative data extracted from the included studies. These results will be narratively summarized and presented in tabular format. Meta-analyses will be conducted, if sufficient data are available and if appropriate: two or more studies with similar disease models for an in vivo pre-clinical efficacy outcome, with the same outcome definition, or two or more studies with a similar in vitro potency assay employed. For continuous endpoints, data will be pooled using the ratio of the weighted means method with inverse variance random effects modeling. The ratio of means effect estimate is preferred because (a) it is unitless and allows pooling of data with different units of measurement within the same outcome, (b) it does not require knowledge of pooled standard deviation, and (c) may be more interpretable to clinicians as compared to the standardized mean difference [46]. Data reported in non-standard format (e.g., mean ± standard error, median, and range) will be converted to mean ± standard deviation. Outcomes will be described at all experimental timepoints reported in the included studies to understand any short- and long-term effects. If possible, the effect of in vitro inhibition on peripheral blood mononuclear cells (PBMCs) in co-culture with MSCs will be pooled to provide a direct, quantitative comparison between the freshly cultured and freshly thawed MSC in vitro potency.

### Subgroup analyses

If possible within the limitations of the data, subgroup meta-analyses will be considered to evaluate sources of high heterogeneity (*I*^*2*^) in our primary meta-analyses and will be hypothesis generating. Potential subgroups of interest include MSC source, route of MSC administration, and MSC preparation and cryopreservation method.

### Knowledge translation

The results of this systematic review will be of interest to a broad audience which includes basic and clinical MSC research scientists, health regulators and funders, and the clinical and public community. We plan to present our findings at future national and international critical care and regenerative medicine meetings. This review will be reported in accordance with the Preferred Reporting Items for Systematic Reviews and Meta-Analyses (PRISMA) guidelines developed for proper reporting of clinical systematic reviews (see Additional File 1).

## Discussion

Our systematic review will comprehensively summarize the pre-clinical research on in vivo surrogate markers of preclinical efficacy and in vitro potency of freshly cultured versus freshly thawed MSCs for acute and chronic inflammatory conditions. Our review will systematically summarize the current evidence base, identify research gaps, and lay the groundwork for future related pre-clinical and clinical research studies.

Our protocol has strengths and limitations. The strengths are that we have designed a systematic, comprehensive, and transparent search to summarize the totality of the evidence. We have also established standardized definitions and methods for the approach to the analysis and interpretation of the included studies. A limitation of our review is that there may be limited opportunity to quantitatively summarize the study outcomes because of the broad pre-clinical model inclusion criteria. However, the broad inclusion criteria provide comprehensiveness to the study and we aim to qualitatively summarize these data for the readership.

## Supplementary information

**Additional file 1.** It provides a completed PRISMA checklist for this systematic review.

**Additional file 2: Table S1.** Draft Search strategy.

## Data Availability

Not applicable.

## Appendix

**Table 3:** Sample of SYRCLE risk of bias tool employed for preclinical studies

Item	Type of bias	Domain	Description of domain	Review authors judgment
1	Selection bias	Sequence generation	Describe the methods used, if any, to generate the allocation sequence in sufficient detail to allow an assessment whether it should produce comparable groups.	Was the allocation sequence adequately generated and applied? (*)
2	Selection bias	Baseline characteristics	Describe all the possible prognostic factors or animal characteristics, if any, that are compared in order to judge whether or not intervention and control groups were similar at the start of the experiment.	Were the groups similar at baseline or were they adjusted for confounders in the analysis?
3	Selection bias	Allocation concealment	Describe the method used to conceal the allocation sequence in sufficient detail to determine whether intervention allocations could have been foreseen before or during enrolment.	Was the allocation adequately concealed? (*)
4	Performance bias	Random housing	Describe all measures used, if any, to house the animals randomly within the animal room.	Were the animals randomly housed during the experiment?
5	Performance bias	Blinding	Describe all measures used, if any, to blind trial caregivers and researchers from knowing which intervention each animal received. Provide any information relating to whether the intended blinding was effective.	Were the caregivers and/or investigators blinded from knowledge which intervention each animal received during the experiment?
6	Detection bias	Random outcome assessment	Describe whether or not animals were selected at random for outcome assessment and which methods to select the animals, if any, were used.	Were animals selected at random for outcome assessment?
7	Detection bias	Blinding	Describe all measures used, if any, to blind outcome assessors from knowing which intervention each animal received. Provide any information relating to whether the intended blinding was effective.	Was the outcome assessor blinded?
8	Attrition bias	Incomplete outcome data	Describe the completeness of outcome data for each main outcome, including attrition and exclusions from the analysis. State whether attrition and exclusions were reported, the numbers in each intervention group (compared with total randomized animals), reasons for attrition or exclusions, and any re-inclusions in analyses for the review.	Were incomplete outcome data adequately addressed? (*)
9	Reporting bias	Selective outcome reporting	State how selective outcome reporting was examined and what was found.	Are reports of the study free of selective outcome reporting? (*)
10	Other	Other sources of bias	State any important concerns about bias not covered by other domains in the tool.	Was the study apparently free of other problems that could result in high risk of bias? (*)

*Items in agreement with the items in the Cochrane Risk of Bias tool

## Abbreviations

MSCs: Mesenchymal stromal/stem cells
PBMCs: Peripheral blood mononuclear cells
ISCT: International Society of Cellular Therapy
ELISA: Enzyme-linked immunosorbent assay
MPCs: Mesenchymal progenitor cells
MNC: Mononuclear cell
IBD: Inflammatory bowel disease
HSC: Hematopoietic stem cells
