# Supplementary material for: Comparison of freshly cultured versus freshly thawed (cryopreserved) mesenchymal stem cells in preclinical in vivo models of inflammation: a protocol for a preclinical systematic review and meta-analysis
Source: Syst Rev. 2020 Aug 19;9:188. doi: 10.1186/s13643-020-01437-z (PMC7437051; doi:10.1186/s13643-020-01437-z)
Supplement: Supplementary file 2 — Additional file 2: Table S1. Draft Search strategy. [file 13643_2020_1437_MOESM2_ESM.docx]

**Table** **S1.** Draft Search strategy.

Database: **Embase Classic+Embase** <1947 to 2019 June 19>, Ovid MEDLINE(R) ALL <1946 to June 18, 2019>

Search Strategy:

--------------------------------------------------------------------------------

1 Mesenchymal Stromal Cells/ (41061)

2 Mesenchymal Stem Cell Transplantation/ (20646)

3 Multipotent Stem Cells/ (8357)

4 (mesenchymal adj3 (cell* or stem or stromal or progenitor or multipotent or bone marrow or adipose or placenta*)).tw. (151237)

5 ((multipotent or multi-potent) adj (stroma* cell* or stem cell* or progenitor cell*)).tw,kw. (5662)

6 (marrow stroma* adj2 cell*).tw,kw. (17559)

7 (mesenchymal or marrow strom* or strom* cell*).kw,kw. (39972)

8 or/1-7 (195002)

9 exp Cryopreservation/ (75083)

10 cryoconserv*.tw,kw. (545)

11 cryopreserv*.tw,kw. (56232)

12 (fresh* adj5 thaw*).tw. (3956)

13 (freez* adj3 thaw*).tw. (26130)

14 (fresh cell* or continuously cultur*).tw,kw. (2822)

15 (thaw* or freez*).kw. (12591)

16 (fresh* adj3 cultur*).tw. (8205)

17 or/9-16 (133981)

18 8 and 17 (2540)

19 18 use medall (931) Medline

20 exp mesenchymal stem cell/ (85689)

21 mesenchymal stem cell transplantation/ (20646)

22 mesenchymal stroma cell/ (11798)

23 (mesenchymal adj3 (cell* or stem or stromal or progenitor or multipotent or bone marrow or adipose or placenta*)).tw. (151237)

24 ((multipotent or multi-potent) adj (stroma* cell* or stem cell* or progenitor cell*)).tw. (5547)

25 (marrow stroma* adj2 cell*).tw. (17188)

26 or/20-25 (184041)

27 cryopreservation/ (62498)

28 cryopreserv*.tw. (54818)

29 (freez* adj3 thaw*).tw. (26130)

30 cryoconserv*.tw. (517)

31 (fresh* adj5 thaw*).tw. (3956)

32 (fresh* adj3 cultur*).tw. (8205)

33 fresh cell*.tw. (2137)

34 continuously cultured.tw. (651)

35 or/27-34 (112796)

36 26 and 35 (2393)

37 conference abstract.pt. (3438015)

38 36 not 37 (1908)

39 38 use emczd (1093) Embase

40 19 or 39 (2024)

41 remove duplicates from 40 (1293)

42 41 use medall (929) Medline

43 41 use emczd (364) Embase

**Web of Science** – June 20, 2019

# 8

1,478

#6 AND #3

Refined by: [excluding] DOCUMENT TYPES: ( MEETING ABSTRACT )

Indexes=SCI-EXPANDED, CPCI-S, CPCI-SSH, ESCI Timespan=1900-2019

# 7

1,542

#6 AND #3

Indexes=SCI-EXPANDED, CPCI-S, CPCI-SSH, ESCI Timespan=1900-2019

# 6

92,964

#5 OR #4

Indexes=SCI-EXPANDED, CPCI-S, CPCI-SSH, ESCI Timespan=1900-2019

# 5

49,605

TI=(mesenchym* stem cell*) OR TI=(stroma* cells*) OR TI=(Multipotent Stem Cell*)

Indexes=SCI-EXPANDED, CPCI-S, CPCI-SSH, ESCI Timespan=1900-2019

# 4

83,235

TS=("Mesenchymal Stem Cell*") OR TS=("Mesenchymal Stem Cell Transplantation") OR TS=("Multipotent Stem Cell*") OR TS=("Mesenchymal Stromal Cell*") OR TS=("marrow stroma* cell*")

Indexes=SCI-EXPANDED, CPCI-S, CPCI-SSH, ESCI Timespan=1900-2019

# 3

99,291

#2 OR #1

Indexes=SCI-EXPANDED, CPCI-S, CPCI-SSH, ESCI Timespan=1900-2019

# 2

21,478

TI=(fresh* thaw*) OR TI=(freez* AND thaw*) OR TI=(cryopreserv*) OR TI=(cryoconserv*)

Indexes=SCI-EXPANDED, CPCI-S, CPCI-SSH, ESCI Timespan=1900-2019

# 1

99,120

TS=(Cryopreserv*) OR TS=(cryoconserv*) OR TS=(fresh* NEAR/3 thaw*) OR TS=(freez* NEAR/3 thaw*) OR TS = (fresh cell*) OR TS=(continuously cultur*) OR TS=(fresh* NEAR/3 cultur)

Indexes=SCI-EXPANDED, CPCI-S, CPCI-SSH, ES

CI Timespan=1900-2019
